# A retrospective pathology study of two Neotropical deer species (1995-2015), Brazil: Marsh deer (*Blastocerus dichotomus*) and brown brocket deer (*Mazama gouazoubira*)

**DOI:** 10.1371/journal.pone.0198670

**Published:** 2018-06-07

**Authors:** Pedro Enrique Navas-Suárez, Josué Díaz-Delgado, Eliana Reiko Matushima, Cintia Maria Fávero, Angélica Maria Sánchez Sarmiento, Carlos Sacristán, Ana Carolina Ewbank, Adriana Marques Joppert, Jose Mauricio Barbanti Duarte, Cinthya dos Santos-Cirqueira, Bruno Cogliati, Leonardo Mesquita, Paulo César Maiorka, José Luiz Catão-Dias

**Affiliations:** 1 Laboratory of Wildlife Comparative Pathology - LAPCOM, School of Veterinary Medicine and Animal Sciences, University of São Paulo, São Paulo, Brazil; 2 Divisão Técnica de Medicina Veterinária e Manejo da Fauna Silvestre (DEPAVE-3), São Paulo, Brazil; 3 Deer Research and Conservation Center (NUPECCE), Department of Animal Science, São Paulo State University, Jaboticabal, São Paulo, Brazil; 4 Instituto Adolfo Lutz (IAL), Centro de Patologia, São Paulo, Brazil; 5 Laboratory of Morphological and Molecular Pathology, School of Veterinary Medicine and Animal Sciences, University of São Paulo, São Paulo, Brazil; 6 Laboratory of Animal Models, School of Veterinary Medicine and Animal Sciences, University of São Paulo, São Paulo, Brazil; US Geological Survey, UNITED STATES

## Abstract

This retrospective study describes the biological and epidemiological aspects, gross and microscopical findings, and most likely causes of death (CD) in two species of Neotropical deer in Brazil. The animals were collected between 1995 and 2015 and represented 75 marsh deer (MD) and 136 brown brocket deer (BBD). Summarized, pneumonia was diagnosed microscopically in 48 MD and 52 BBD; 76 deer suffered trauma, involving dog attack (14 BBD) and vehicle-collision (14 BBD). Pulmonary edema (50 MD; 55 BBD) and congestion (57 MD; 78 BBD) were the most common findings for both species. Additionally, we diagnosed ruminal and myocardial mycosis in MD and BBD, respectively; ovarian dysgerminoma and pancreatic trematodiasis in BBD; and lesions suggestive of malignant catarrhal fever and orbiviral hemorrhagic disease in both species. The main CD in MD was: respiratory (41/75), alimentary, nutritional, trauma and euthanasia (3/75 each). Correspondingly, in BBD were: trauma (34/131), respiratory (30/131) and euthanasia (9/131). Respiratory disease was often defined by pulmonary edema and pneumonia. We provide evidence that respiratory disease, mainly pneumonia, is a critical pathological process in these Neotropical deer species. Although no etiological agents were identified, there is evidence of bacterial and viral involvement. Our results show trauma, mainly anthropogenic, as a common ailment in BBD. We propose to prioritize respiratory disease in future research focused on South American deer health aspects. We believe anthropogenic trauma may be a primary threat for populations of BBD.

## Introduction

In wildlife, diseases may lead to population decline [1–3]. Some deer species, mainly the white-tailed deer (WTD; *Odocoileus virginianus*), have been extensively studied, with reports of infectious (prion, viral, bacterial, fungal, parasitic) and non-infectious diseases (e.g., toxicities, nutritional and metabolic disorders, neoplasia) [4–5]. Wildlife plays a crucial role in the epidemiology of emerging human and livestock infectious agents. It is estimated that more than 70% of these agents can infect multiple species [6–7]. Zoonotic and non-zoonotic examples of infectious diseases in wild ruminants with major socio-economic implications include: foot and mouth disease (FMD) in WTD [8–9]; bluetongue (BT) in WTD and red deer (*Cervus elaphus*) [10–11]; bovine viral diarrhea (BVD) in WTD [12–13]; chronic wasting disease in WTD [14]; and brucellosis in several North American deer species [15].

Brazilian livestock production is the second largest in the world (approximately 200 million animals) and holds a significant percentage of the gross domestic product [16]. Brazilian grazing system is mainly extensive (approximately 93% in all states), which favors direct and indirect contact between cattle and wildlife [17]. Diseases introduced by livestock (e.g., FMD, brucellosis, babesiosis and several ecto- and endoparasitoses) are considered threats to the conservation of the eight-deer species currently found in Brazil [18]. However, the susceptibility of such species to the majority of livestock pathogens is poorly understood. Health studies in South American deer species have mainly focused on serological surveys, e.g., brown brocket deer (BBD; *Mazama gouazoubira*) in Bolivia [19], Argentinean Pampas deer (*Ozotoceros bezoarticus*) [20], and several studies of free-ranging and captive Brazilian deer populations [21–26].

Specifically, the marsh deer (MD; *Blastocerus dichotomus*) and the BBD, are included in the Brazilian National Action Plan for Conservation of Endangered South American Deer [27]. The former is the largest deer species in South America and one of the largest mammals in Brazil [27]. It is regarded as vulnerable by the Convention on International Trade in Endangered Species of Wild Fauna and Flora (CITES; Appendix I) and the International Union for Conservation of Nature and Natural Resources (IUCN) [18]. The BBD is the most common deer in Brazil. Although it is not included in the CITES and is overall classified as of least concern in the IUCN Red List of Threatened Species, the BBD is considered “Vulnerable” and “Endangered”, in Rio Grande and Rio de Janeiro States, respectively, mainly because of habitat destruction and hunting [18].

Health monitoring studies of free-ranging deer populations and the livestock-wildlife interface are required in order to further clarify the challenges involved in these wild species’ conservation. This study aimed to describe retrospectively biological and epidemiological aspects, gross and microscopic findings and possible causes of death of two Neotropical deer species in Brazil, MD and BBD.

## Materials and methods

### Collection of epidemiological and biological data

Retrospective data on epidemiological (season, circumstances of death, history of trauma, ectoparasitosis) and biological variables (sex, age, body condition) from 75 MD and 131 BBD were retrieved from the electronic database, historically maintained by different institutions and regions of São Paulo state, Brazil. These data and preserved tissues from postmortem analyses were gathered over a 21-year period (1995–2015) at the Laboratory of Wildlife Comparative Pathology (LAPCOM), Department of Pathology, School of Veterinary Medicine and Animal Science, University of São Paulo (VPT-FMVZ-USP). For some cases epidemiological and/or necropsy data were not available (these cases are labeled ‘NR’ in S2 Table).

This study was carried out in compliance with the System Authorization and Information on Biodiversity (SISBIO) of the Brazilian Institute of Environment and Renewable Natural Resources (IBAMA) (license number: 47858–1) and was approved by the Ethics Committee on Animal Use (CEUA) of the School of Veterinary Medicine and Animal Science—University of São Paulo (FMVZ-USP) (protocol number: 4271090215). All animals were dead at the time of submission to necropsy; no animals were euthanized in this study.

### Microscopic and histochemical analyses

Representative tissue samples collected during necropsy were fixed in 10% neutral buffered formalin, embedded in paraffin-wax (FFPE), processed as routine, sectioned at 5 μm and stained with hematoxylin and eosin for light microscopic examination. Special histochemical stains including Congo red for amyloid; Gram for bacteria; Grocott-methenamine silver (GMS) for fungi; luxol fast blue (LFB) for myelin; Masson’s trichrome (MT) for collagen and muscular fibers; periodic acid-Schiff (PAS) for fungi; and Von Kossa for calcium [28], were used. There was certain heterogeneity in tissue samples available for histopathology (the tissues evaluated by species is recorded in S1 Table). All microscopic findings were categorized on a five-degree severity scale (mild, mild-moderate, moderate, moderate-severe, severe). When possible, the etiology was listed as: bacterial, viral, fungal and parasitic. Morphologic identification of metazoan and protozoan parasites in tissue sections was based on published guides [29–30].

### Immunohistochemical analysis

Immunohistochemical (IHC) analyses were performed in the IHC laboratory at the Adolfo Lutz Institute and the laboratory of Animal Models and Morphological and Molecular Pathology (VPT-FMVZ-USP). For IHC, FFPE tissues were sectioned at 4 μm. IHC methodology details on antibodies dilutions and incubation times are recorded in Table 1. The heat-induced epitope retrieval (HIER) was performed in a water bath at 98°C for 20 min or in an electric pressure cooker (110 V, 60 Hz) for 15 mins, using citrate (pH 6.0) and/or EDTA (pH 8.0) buffer. The blocking of endogenous peroxidase activity was performed in methanolic solution of hydrogen peroxide (3%). All primary antibodies were incubated in a humid chamber at 4°C for 16–18 hours (overnight). The binding between antigens and antibodies was visualized using diaminobenzidine (Vectastain^®^; Vector laboratories, Burlingame, CA, USA) per manufacturer’s instructions, followed by slight counterstain with Harry’s hematoxylin. In negative controls, primary antibodies were substituted by homologous non-immune sera. Sections of normal deer cerebrum and kidney were used as positive controls.

**Table 1:** Antibody reagents, antigen retrieval and detection systems used in immunohistochemistry.

Antibody (clone)	Dilution	Pretreatment (HIER)	Detection System	Manufacturer
GFAP (polyclonal)	1:400	Water bath for 20 min (98°C, citrate buffer, pH 6.0)	Dako LSAB^®^ detection kit	Dako, Agilent Pathology Solutions, Santa Clara, CA, USA
Myoglobin (polyclonal)	1:45,000	Water bath for 20 min (98°C, citrate buffer, pH 6.0)	Dako LSAB^®^ detection kit	*in house* [Adolfo Lutz Institute]
CD3 (polyclonal)	1:2,000	Water bath for 20 min (98°C, citrate buffer, pH 6.0)	Dako LSAB^®^ detection kit	Dako, Agilent Pathology Solutions, Santa Clara, CA, USA
CD20 (polyclonal)	1:1000	Pressure cooker, citrate buffer, pH 6.0	REVEAL Polyvalent HRP-DAB Detection System, Spring Bioscience	Thermo Fisher Scientific, Waltham, MS, USA
Vimentin (V9)	1:1000	Pressure cooker, citrate buffer, pH 6.0	REVEAL Polyvalent HRP-DAB Detection System, Spring Bioscience	Dako, Agilent Pathology Solutions, Santa Clara, CA, USA
S100 (polyclonal)	1:2000	Pressure cooker, citrate buffer, pH 6.0	REVEAL Polyvalent HRP-DAB Detection System, Spring Bioscience	Dako, Agilent Pathology Solutions, Santa Clara, CA, USA
Pan-Cytokeratin (AE1/AE3)	1:1000	Pressure cooker, citrate buffer, pH 6.0	REVEAL Polyvalent HRP-DAB Detection System, Spring Bioscience	Dako, Agilent Pathology Solutions, Santa Clara, CA, USA
Synaptophysin (SY38)	1:2000	Pressure cooker, citrate buffer, pH 6.0	REVEAL Polyvalent HRP-DAB Detection System, Spring Bioscience	Dako, Agilent Pathology Solutions, Santa Clara, CA, USA

### Molecular analysis

Polymerase chain reaction (PCR) analysis was performed in ten sections (10 μm-thick) of selected FFPE tissues. Deparaffinization was achieved by consecutive xylol and phosphate-buffered saline washes. DNA extraction was carried out with the aid of 200 μL of tissue suspension obtained by the proteinase K/phenol-chloroform method [31]. The integrity of the extracted DNA was evaluated by PCR analysis for the β-actin gene [32]. Furthermore, PCR analysis using primers described for fungal ITS amplification was conducted at a melting temperature of 55°C [33–34]. Detection of *Ovine herpesvirus* 2 (OvHV-2) and *Alcelaphine herpesvirus* 1 (AlHV-1) was performed according to OIE [35] and Van Devanter et al. [36] protocols, at melting temperatures of 45°C and 60°C, respectively. Finally, PCR products were submitted to electrophoresis in agarose gel (1.5%) stained with SYBER safe (Thermo Fisher Scientific, Waltham, MS, USA). Samples of goat and sheep herpesvirus positive samples were used as positive controls. *Paracoccidioides brasiliensis* DNA was used as positive control for fungi. Positive samples were confirmed by direct sequencing. The obtained sequences were then aligned with similar ones available at GenBank with the aid of the CLUSTAL/W method, followed by the p-distance analysis by the MEGA 7 program to determine the identity percentage.

## Results

Epidemiological and biological data, gross and microscopic findings, and most probable causes of death are recorded in S2 Table. Necropsy reports were available for 70 of 75 MD and 90 of 131 BBD.

Marsh deer (n = 75) were of various sex: 40 (53.3%) female, 31 (41.3%) male, 4 undetermined; and age: 48 (64%) adult, 12 (16%) juvenile, 12 (16%) fawn, 3 undetermined. Forty (53.3%), 12 (16%) and 19 (25.3%) animals were in good, regular and poor body condition, respectively. In four cases the body condition was unknown. Brown brocket deer (n = 131) were of various sex: 54 (41.2%) female, 60 (45.8%) male, 17 undetermined; and age: 60 (45.8%) adults, 28 (21.4%) juveniles, 16 (12.2%) fawns, and 27 undetermined. Sixty-nine (52.7%), 15 (11.5%) and 13 (9.9%) animals were in good, regular and poor body condition, respectively. The body condition was undetermined in 34 cases.

### Gross findings

The main gross findings are summarized in Table 2.

**Table 2:** Main gross findings in MD and BBD.

Gross findings	MD (n = 70)	BBD (n = 90)
**Alimentary tract, liver and pancreas**		
*Congestion*	22 (31.4%)	25 (27.8%)
*Hemorrhage*	16 (22.9%)	11 (12.2%)
*Inflammatory process*	27 (38.5%)	18 (20%)
*Hepatomegaly*	9 (12.9%)	16 (17.8%)
*NMA*	13 (18.6%)	35 (38.9%)
**Cardiovascular system**		
*Hemorrhage*	17 (24.3%)	13 (14.4%)
*Hydropericardium*	17 (24.3%)	8 (8.9%)
*Inflammatory process*	10 (14.3%)	3 (3.3%)
*NMA*	24 (34.3%)	59 (65.6%)
**Endocrine system**		
*Adrenal congestion*	3 (4.3%)	2 (2.2%)
*Adrenal gland hemorrhage*	1 (1.4%)	1 (1.1%)
*Adrenomegaly*	4 (5.7%)	1 (1.1%)
*Adrenal gland edema*	3 (4.3%)	None
*NMA*	60 (85.7%)	82 (91.1%)
**Hematopoietic system**		
*Congestion*	8 (11.4%)	8 (8.9%)
*Hemorrhage*	3 (4.3%)	3 (3.3%)
*Splenomegaly*	14 (20.0%)	12 (13.3%)
*Inflammatory process*	1 (1.4%)	1 (1.1%)
*NMA*	43 (61.4%)	63 (70.0%)
**Integumentary system**		
*Hematoma*	6 (8.6%)	27 (30%)
*Skin laceration*	15 (21.4%)	31 (34.4%)
*Ectoparasites*	11 (15.7%)	23 (25.6%)
*NMA*	24 (34.3%)	18 (20%)
**Musculoskeletal system**		
*Bone fractures*	10 (14.3%)	26 (28.9%)
*Luxation*	5 (7.1%)	1 (1.1%)
*Rhabdomyolysis*	9 (12.9%)	16 (17.8%)
*NMA*	41 (58.6%)	2 (2.2%)
**Nervous system**		
*Cortex congestion*	4 (5.7%)	10 (11.16%)
*Cerebral hemorrhage*	None	4 (4.4%)
*Hematomyelia*	1 (1.4%)	None
*Spinal compression*	1 (1.4%)	None
*Brain hematoma*	None	2 (2.2%)
*NMA*	62 (88.6%)	71 (78.9%)
**Respiratory system**		
*Edema*	41 (58.6%)	48 (53.3%)
*Congestion*	28 (40.0%)	44 (48.9%)
*Pneumonic process*	24 (34.3%)	12 (13.3%)
*Hemorrhage*	14 (20.0%)	13 (14.4%)
*Emphysema*	8 (11.4%)	5 (5.6%)
*NMA*	8 (11.4%)	17 (18.9%)
**Urogenital System**		
*Congestion*	13 (18.6%)	14 (15.6%)
*Hemorrhage*	18 (25.7%)	11 (12.2%)
*Inflammatory process*	17 (24.3%)	26 (28.9%)
*Degenerative changes*	16 (22.9%)	None
*NMA*	21 (30%)	3 (3.3%)

NMA: No macroscopic alterations.

Trauma was reported in 36.9% cases (11 MD, 65 BBD), mainly associated with vehicle-collision (15 BBD), dog attack (14 BBD), self-induced (4 MD, 1 BBD), intraspecific interaction (2 BBD) and unknown (7 MD, 33 BBD). BBD affected by vehicle-collision involved: nine females and six males; 13 adults, one juvenile an one of undetermined age; 12 animals in good body condition, one in regular, and one in poor and one undetermined. BBD attacked by dogs included: ten females and four males; six adults, five juveniles, two fawns and one undetermined; 13 animals in good body condition and one in regular.

### Microscopic findings

The main microscopic findings are recorded in Table 3.

**Table 3:** Main microscopic findings in MD and BBD.

Histopathological findings	MD (Na/Ne)	BBD (Na/Ne)
**Alimentary tract, liver and pancreas**		
*Colitis*	3/3	5/17
*Enteritis*	4/18	6/46
*Glossitis*	4/15	4/29
*Hepatic hemorrhage*	23/59	20/92
*Hepatitis*	29/59	37/92
*Rumenitis*	1/14	2/52
*Hepatocellular steatosis*	26/59	50/92
**Cardiovascular system**		
*Cardiac congestion*	2/41	6/79
*Myocardial hemorrhage*	8/41	11/79
*Myocarditis*	None	3/79
*Sarcocystis* sp. cyst	None	7/79
**Endocrine System**		
*Congestion of adrenal glands*	6 / 15	10 / 32
*Hemorrhage of adrenal glands*	6 / 15	18 / 32
*Adrenalitis*	1 / 15	3 / 32
**Hematopoietic system**		
*Splenic white pulp depletion*	20 / 44	27 / 83
*Splenic hemosiderosis*	10 / 44	7 / 83
*Lymph node congestion*	3 / 13	3 / 19
*Lymph node hemorrhage*	1 / 13	5 / 19
**Integumentary system**		
*Myiasis*	None	1 / 19
*Dermatitis*	1 / 3	6 / 19
**Musculoskeletal system**		
*Myositis*	9 / 31	9 / 44
*Muscle necrosis*	8 / 31	16 / 44
*Muscle hemorrhage*	7 / 31	11 / 44
Inflammation of diaphragm	1 / 31	None
*Sarcocystis sp*. *cyst*	3 / 31	7 / 44
**Nervous system**		
*Cerebral edema*	3 / 10	5 / 55
*Cerebral congestion*	1 / 10	16 / 55
*Cerebral hemorrhage*	1 / 10	4 / 55
*Meningoencephalitis*	None	1 / 55
**Respiratory system**		
*Pneumonia*	48 / 70	52 / 95
*Pulmonary congestion*	57 / 70	78 / 95
*Pulmonary edema*	50 / 70	55 / 95
*Pulmonary hemorrhage*	19 / 70	29 / 95
*Tracheal hemorrhage*	4 / 9	3 / 14
**Urogenital System**		
*Acute tubular necrosis*	9 / 66	2 / 88
*Cystitis*	1 / 16	1 / 19
*Glomerulonephritis*	7 / 66	19 / 88
*Interstitial nephritis*	17 / 66	9 / 88
*Renal congestion*	46 / 66	62 / 88
*Renal hemorrhage*	8 / 66	8 / 88
*Proteinosis*	7 / 66	11 / 88
*Tubular degeneration*	26 / 66	17 / 88

Na = number of affected individuals, Ne = number of examined individuals.

### Alimentary tract, liver and pancreas

Gross findings: Inflammatory disease processes involving the digestive tract were grossly noted in 28.1% cases. By decreasing prevalence, inflammation affected the small intestine (catarrhal [3 BBD], hemorrhagic [8 MD, 3 BBD], necrotizing [2 MD], parasitic [2 MD]), liver (parasitic [1 MD], abscedative [1 BBD], necrotic [1 MD]), abomasum (hemorrhagic [2 MD], parasitic [1 MD]), stomatitis (2 MD), colon (hemorrhagic [2 BBD], catarrhal [1 BBD]) and esophagus (gongylonemiasis; 1 MD).

**Fig 1:**
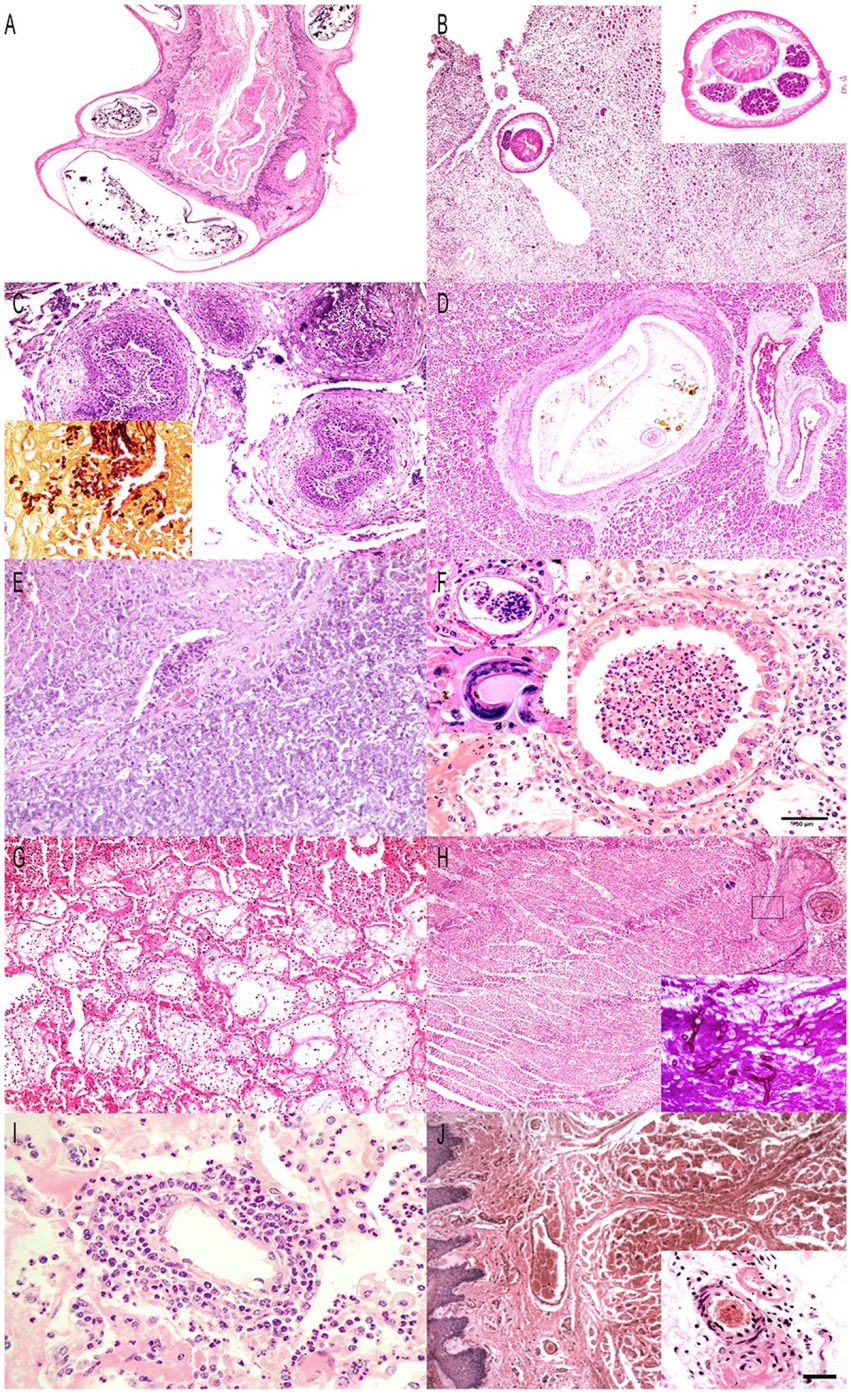
Microscopic inflammatory findings in BBD and MD. A) Esophagus, MD, adult, female (BD-37). Multiple transverse and focal oblique sections of nematodes compatible with *Gongylonema* within mildly hyperplastic esophageal epithelium. H&E. (20x). B) Diaphragm, MD, adult, male (BD-22). Marked, extensive, chronic myositis with intralesional unidentified adult nematode. H&E. (4x). Inset: Cross-section of adult female ascarid nematode with cuticle having lateral alae, intestine with large columnar epithelium and apical brush border, pseudocoelum with eosinophilic fluid, and ovaries. C) Ruminal papillae, MD, fawn, male (BD-010). Multiple ruminal papillae are infiltrated by neutrophils and the overlying epithelium presents degeneration and necrosis and superficial bacteria. H&E (10x). Inset: Numerous yeasts and pseudohyphae are present within an inflamed ruminal papilla. GMS (40x). D) Pancreas, BBD, adult, female (MG-116). Multiple adult trematodes compatible with *Eurythrema* sp., expand and obliterate the lumen of an intrapancreatic duct. H&E (10x). E) Liver, BBD, adult, male (MG-120). Expanding the hepatic parenchyma is a nodular, un-encapsulated, densely cellular and mildly infiltrative neoplasm composed of polygonal neoplastic cells arranged in cords supported by a delicate fibrovascular stroma. H&E (10x). F) Lung, BBD, adult, female (MG-027). Suppurative exudate fills in the lumen of a bronchiole and adjacent alveoli. H&E. (20x). Left upper corner inset: nematode egg within alveolar inflammatory focus. H&E. (40x). Left middle inset: nematode larvae within edematous alveolus. H&E. (40x). G) Lung, MD, juvenile, female (BD-34). Alveolar septa are necrotic, distorted and filled with edema, fibrin, hemorrhage and degenerate neutrophils. H&E. (10x). H) Heart, MG, juvenile, male (MG-014). Myocarditis with cardiomyocyte necrosis associated with focally obliterative fungal thromboembolus (asterisk). Inset: Hyphal angioinvasion (squared area in main Fig 2H). PAS (10x). I) Lung, MD, adult, male (BD-40). Moderate numbers of lymphocytes, neutrophils and few plasma cells infiltrate and distort the vascular wall. The adjacent alveoli are filled with edema and suppurative exudate. H&E. (20x). J) Tongue, BBD, adult, male (MG-013). Focal acute hemorrhage in intrinsic glossal musculature and submucosa. H&E (10x). Inset: Focal fibrinoid vascular necrosis. Few lymphocytes and plasma cells infiltrate the perivascular collagen fibers of an adjacent arteriole. H&E. (20x).

Microscopic findings: Inflammation of the digestive tract was observed in the tongue (4 MD, 4 BBD), rumen (1 MD, 2 BBD), reticulum (1 MD, 1 BBD), small intestine (4 MD, 6 BBD), large intestine (3 MD; 5 BBD) and liver (29 MD, 37 BBD). Tonsillitis was classified as nonsuppurative (2 MD, 2 BBD), necrotizing (2 BBD), suppurative (1 BBD) and glossal abscess (1 MD). One MD presented multiple intraepithelial nematodes compatible with *Gongylonema* sp. in the esophagus (Fig 1A). One BBD presented diaphragm myositis with intralesional nematodes compatible with ascarids (Fig 1A). Rumenitis (Fig 1C) was typically characterized by infiltration of neutrophils, lymphocytes and plasma cells, necrosis of papillae and varying degrees of epithelial ulceration and hemorrhage. The etiology was determined only in one case involving a male fawn MD with marked necrohemorrhagic and perforating fungal rumenitis that lead to septic peritonitis and bloating. PCR sequencing from FFPE rumen tissue sections yielded a 577 bp sequence (excluding primers) with 99.8% nucleotide identity to the *Aureobasidium pullulans* fungus (JF439462). The novel sequence was submitted to GenBank under the accession number MG547431.

Hepatitis (29 MD; 37 BBD) was classified into: portal (21 MD, 22 BBD; typically composed of lymphoplasmacytic infiltrates, lacking active necrosis) or diffuse (8 MD; 15 BBD). One BBD had *Eurythrema* sp. pancreatic ductitis (Fig 1D). Hepatic hemorrhage was classified as: diffuse (18 MD; 5 BBD), midzonal (2 MD; 7BBD), midzonal-centrilobular (3 MD; 4 BBD), and portal (2 MD). Congestion was typically diffuse. Hepatic steatosis was a relatively common finding, including macrovesicular (17 MD; 40 BBD), microvesicular (5 MD); and combined (4 MD; 10BBD) patterns. In one BBD, expanding the hepatic parenchyma, there was a poorly differentiated primary hepatic neuroendocrine neoplasm (Fig 1E) with rare lymphovascular invasion and negative immunolabeling for chromogranin, synaptophysin, AE1/AE3, CD3 and CD20.

### Musculoskeletal system

**Fig 2:**
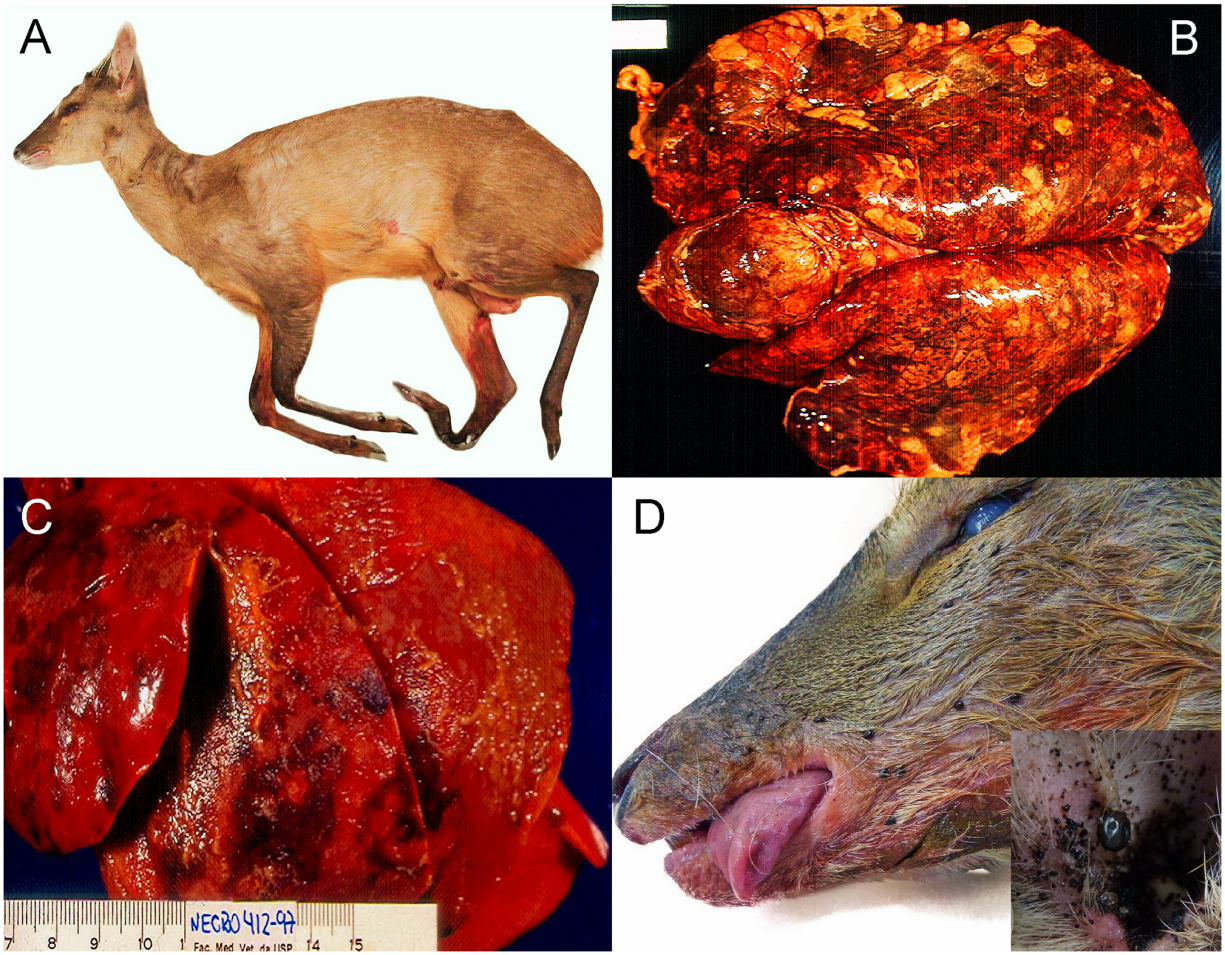
Gross findings in brown brocket deer. A) Whole body, BBD, adult, male (MG-135). Distal right metatarsal fracture due to vehicle collision. B) Lung, BBD, adult, female (MG-045). Bilateral, lobar pneumonia with hemorrhage. C) Lung, BBD, juvenile, male (MG-039). Suppurative bronchopneumonia with hemorrhage and fibrinous pleuritis. D) Left side of rostrum, BBD, female, adult (MG-125). Multiple ticks are on the skin of the cheek region. Inset: Argasidae ticks in the external ear canal.

Gross findings: Fractures (Fig 2A) were described in 22.5% of cases, mainly involving the skull (7 MD; 5 BBD); hindlimbs (10 BBD); ribcage (4 MD; 4 BBD); pelvic girdle (1 MD; 5 BBD); fore limbs (1 MD; 4 BBD), and vertebral column (1 MD; 3 BBD). Traumata were often associated with skin lacerations (28.8%). These were observed on limbs (5 MD; 2 BBD), head (1 BBD), thorax (1 MD), or in two or more anatomical regions (9 MD; 27 BBD).

Microscopic findings: Myositis, characterized by neutrophilic infiltrates, myofiber degeneration and necrosis, and hemorrhage was noted in 9 MD and 9 BBD. Cortical adrenalitis, predominately lymphocytic with occasional hemorrhage and necrosis, was observed in one MD and three BBD. In most of these cases, an etiology was not readily apparent, except for one case where adrenalitis was associated with bacterial sepsis. Intrasarcoplasmic *Sarcocystis* sp. cysts were observed in the heart (7 BBD); tongue (1 BBD); esophagus (10 BBD); and axial and appendicular skeletal myofibers (3 MD; 7 BBD). None of these cases had an inflammatory response to the protozoan cysts.

### Respiratory system

Gross findings: Pneumonic (Fig 2B and 2C) processes (22.5%) were grossly classified as: bronchopneumonia (8 MD; 1 BBD), aspiration pneumonia (2 MD; 3 BBD), pleuropneumonia (4 MD). Some animals (10MD; 8BBD) had ‘undefined pneumonia’.

Microscopic findings: Bronchopneumonia (20 MD; 28 BBD) was classified as suppurative (Fig 1F), fibrinosuppurative and fibrinonecrotic (Fig 1G). Intralesional bacteria were observed in six MD. Verminous pneumonia was seen in 14 MD. Metastrongyle nematodes (Fig 1F) were found in four MD with bronchopneumonia having granulocytic and histiocytic (with multinucleated giant cells) infiltrates, phagocytosis of larvae, marked congestion and edema, and rare emphysema and fibrosis. Histological features of eggs and adult nematodes were compatible with *Dictyocaulus* sp. Additionally, hydatid cyst-like debris surrounded by pleocellular inflammatory infiltrates were observed in two BBD.

Lung congestion (57 MD; 78 BBD) was the most common hemodynamic disorder in both species, followed by edema (50 MD; 55 BBD) and hemorrhage (19 MD; 29 BBD). Thrombosis was observed in three MD and one BBD. Congestion was present in more than 80% of the animals, and was classified as severe in 15 MD and 8 BBD. Severe edema was recorded in 14 MD and 10 BBD. Hemorrhage was severe in three MD and three BBD; in two BBD, hemorrhage was linked to extensive coagulative necrosis (acute infarction).

### Urogenital system

Gross findings: Inflammation of the urogenital system was reported in 43 (26.9%) cases. Renal inflammation was macroscopically classified as: interstitial (4 MD; 21BBD), necrotic (2 BBD), and undefined (7 MD). Undetermined metritis was seen in 6 MD and 2 BBD. Other less frequent processes were: undetermined cystitis (1MD; 1BBD) and necrotic orchitis (1BBD).

Microscopic findings: Inflammatory lesions in the kidneys were classified as: lymphoplasmacytic and histiocytic interstitial nephritis and/or tubulointerstitial nephritis (25 MD; 21 BBD), and glomerulonephritis (membranous [2 MD; 2 BBD] as highlighted by PAS; and proliferative [3 MD; 14 BBD]). Two cases also presented pyelonephritis, necrosis and hemorrhage. Renal congestion was classified into: cortico-medullary (22 MD; 35 BBD), medullary (19 MD; 21 BBD), and cortical (5 MD; 21 BBD). Acute tubular necrosis with intratubular necrotic casts was seen in 9 MD and 2 BBD. All these cases had clinical histories compatible with acute stress and capture myopathy. Intratubular and/or tubuloepithelial myoglobin was noted in all animals. In one female BBD, the normal ovarian tissue was replaced by a poorly demarcated, unencapsulated, densely cellular neoplasm with cytomorphological features compatible with a dysgerminoma. IHC analysis showed neoplastic cells to be vimentin and S-100 positive.

### Cardiovascular system

Microscopic findings: Myocarditis was observed in three BBD: two adults with focal lymphocytic infiltration with hemorrhage and necrosis of the epicardium and one juvenile male with marked fungal myocarditis (Fig 1H). The latter was characterized by thrombosis, severe necrosis and numerous 10–20 μm wide intravascular, transmural and intramyocardial thin-septated hyphae with acute-angle branching and occasional conidia (PAS- and GMS-positive), marked embolic pneumonia with intracapillary bacterial clusters, and marked, diffuse necrotizing esophagitis with hemorrhage. PCR analysis of FFPE heart tissue sections yielded a 530 bp sequence (excluding primers) presenting close nucleotide identity (99.6%) to *Phaeosphaeria* sp. sequences (KC785561 and HQ631018). The novel sequence was submitted to GenBank under the accession number MG547569. Cardiovascular hemorrhage (8MD; 11BBD), and congestion (2MD; 6BBD) were mainly found in the myocardium and pericardium.

### Nervous system

Microscopic findings: Inflammation of the central nervous system (CNS) was observed in one juvenile male BBD with a focally expansive, chronic submeningeal abscess. Microscopically, there was severe, diffuse meningeal and superficial parenchymal lymphohistiocytic and neutrophilic infiltrates and necrosis, rimmed by fibroplasia, astrocytosis and astrogliosis. Glial fibrillary acidic protein (GFAP) immunolabeling evidenced adjacent glial scarring. Cerebral congestion (1 MD; 16 BBD), edema (3 MD; 5 BBD), and hemorrhage (1 MD; 4 BBD) were the main findings in the brain; the latter was observed in cases of trauma.

### Integumentary system

Gross findings: Hematomas (20.6%) were found in the cervical region (1 MD; 2 BBD); thorax (2 BBD); forelimbs (2 BBD); skull (1 MD); jaw (1 BBD); abdomen (1 BBD); hind limbs (1 BBD); and multicentric (4 MD; 18 BBD), typically associated with fractures and lacerations. Other less frequent findings in animals that had trauma were: tibiotarsal (3 MD; 1 BBD) and atlanto-axial joint luxation (2 MD); hematomyelia (1 MD), and spinal cord compression (1 MD). Ectoparasitic infestation (21.3%) were less commonly observed (Fig 2D) (ticks [9 MD; 20 BBD]; myiasis (2 MD; 2 BBD); fleas [2 MD; 1 BBD]; and lice [1 BBD]).

### Endocrine system

Microscopic findings: The adrenal glands presented moderate or severe cortical and/or pericapsular hemorrhage (6 MD, 18 BBD).

### Hematopoietic system

Microscopic findings: Splenic hemosiderosis was observed in 10 MD. Splenic lymphoid depletion (20 MD; 27 BBD) was moderate to marked in 16 MD and 26 BBD.

### Multisystemic lesions

Gross findings: Hemorrhages were observed in the lungs (14MD; 13BBD), kidneys (13MD; 3BBD), liver (12MD; 4BBD), urinary bladder (2MD; 6BBD), forestomachs (2MD; 5BBD), intestines (2MD; 4BBD), testicles (3MD; 1BBD), pancreas (1MD), and uterus (1BBD). Congestion of the digestive system involved the liver (11MD; 24BBD), intestines (8MD; 3BBD), and forestomachs (4MD; 2BBD).

Microscopic findings: Perivasculitis (Fig 1I) was observed in 10 MD and three BBD. In all MD cases, perivasculitis foci mainly had lymphoplasmacytic infiltrates; however, four specimens also presented neutrophils. On these, concomitant fibrinosuppurative bronchopneumonia was noted. All BBD cases presented infiltrates composed of lymphocytes, plasma cells and histiocytes. Due to previous reports of MCF in Brazilian *Mazama* sp., we performed molecular tests for ruminant gammaherpesviruses (AlHV-1 and OvHV-2) identification. The tests were negative.

Eight (2 MD; 6 BBD) animals had lesions suggestive of deer orbiviral hemorrhagic disease with varying degrees of facial and submandibular edema, tongue swelling, generalized congestion and petechiae. These hemodynamic changes were histologically associated with multisystemic congestion and hemorrhage, systemic microangiopathy including vascular fibrinoid necrosis in small and medium-caliber blood vessels and capillaries in the tongue, adrenal glands, heart and lungs (Fig 1J). Systemic thrombosis was diagnosed in one BBD with severe fungal myocarditis.

### Causes of death

Overall, animals with fatal trauma were overrepresented, mainly involving BBD and free-ranging animals (Table 4). Respiratory system was a significant cause of death, being more frequent in MD in comparison to BBD.

**Table 4:** Causes of death in MD and BBD.

CAUSES OF DEATH	SPECIES	
	MD (N = 75)	BBD (N = 131)
*Capture myopathy*	None	8 (6.1%)
*Cardiovascular*	1 (1.3%)	7 (5.3%)
*Dehydration*	None	1 (0.8%)
*Digestive*	4 (5.3%)	None
*Nervous*	None	1 (0.8%)
*Respiratory*	30 (40.0%)	20(15.3%)
*Sepsis*	None	1 (0.8%)
*Starvation*	9 (12.0%)	6 (4.6%)
*Trauma*	12 (16.0%)	48 (36.6%)
*Urinary*	1 (1.3%)	None
*Not evident*	13 (17.3%)	28 (21.4%)
*Advanced autolysis*	5 (6.7%)	11 (8.4%)

### Marsh deer

The most probable causes of death (CD) in MD were: respiratory (30/75), followed by not evident (13/75), trauma (12/75), starvation (9/75). Digestive (4/75), circulatory (1/75) and urinary (1/75) were the least frequent CD, and finally, CD was undetermined by autolysis in 5 cases (Table 4). The most likely CD in adults (n = 22); was: respiratory (pneumonia [14/22], pulmonary edema [7/22], and pulmonary infarct [1/22]); trauma (n = 9; euthanasia [3/9], self-induced [2/9], and unknown cause [4/9]); starvation (n = 5); and urinary (n = 1; as a consequence of suppurative pyelonephritis [1/1]). A CD was not evident in seven cases and undetermined by autolysis in four. In juveniles, the most likely CD was: respiratory (n = 6; pulmonary edema [4/6], pneumonia [1/6], and compression by bloating [1/6]); trauma (n = 2; self-induced [1/2], and unknown [1/2]); starvation (n = 1); digestive (n = 1; colitis); and circulatory (n = 1, hemorrhagic disease). A CD was undetermined by autolysis in one. In fawns, most likely CD was: digestive (n = 3; hepatopathy [2/3], and mycotic rumenitis [1/3]); starvation (n = 3); respiratory (n = 2; involving pulmonary edema [1/2] and pneumonia [1/2]); trauma (n = 1). A CD was not evident in three cases and undetermined by autolysis in one.

### Brown brocket deer

The most probable CD in BBD were: trauma (48/131), not evident (28/131), respiratory (20/131), capture myopathy (8/131), circulatory (7/131), and starvation (6/131), followed by dehydration (1/131), sepsis (1/131) and nervous (1/131). The CD was undetermined by autolysis in 11 cases (Table 4).

In adults, the most probable CD was trauma (n = 33; vehicle-collision [13/33], dog-attack [5/33], self-induced [1/33], and unknown [14/33]); respiratory (n = 6; pneumonia [4/6], pulmonary edema [1/6], and pulmonary infarct [1/6]); capture myopathy (n = 3); circulatory (n = 3; by hemorrhagic disease), starvation (n = 2); and dehydration (n = 1). A CD was not evident and undetermined by autolysis in six cases each. In juveniles, the most likely CD was: trauma (n = 10; dog attack [3/10], vehicle-collision [1/10], intraspecific [1/10], and unknown (5/10]); respiratory (n = 6; pulmonary edema [3/6], and pneumonia [3/6]); capture myopathy (n = 3); cardiovascular (n = 1; by mycotic myocarditis); sepsis (n = 1; by peritonitis); nervous (n = 1; by cerebral abscess); and starvation (n = 1). A CD was not evident in three cases and was undetermined in two. In fawns, the most probable CD was trauma (n = 4; dog-attack [1/4], and unknown [3/4]); starvation (n = 3); capture myopathy (n = 1); and respiratory (n = 1). A CD was not evident in six cases.

## Discussion

Herein, we provide retrospective survey results on biological and epidemiological aspects, gross and microscopic pathologic findings, and most probable CD in two species of Neotropical deer in Brazil.

The estimated population density of MD and BBD in Brazil is 0.50–0.54 deer/km^2^ [37] and 7 deer/km^2^ [38], respectively. This could explain the overrepresentation of BBD in this study. Furthermore, cases with good body condition mainly involved free-ranging animals, in dry period and/or with historical trauma. Environmental changes [39], increase in population density [40] and endoparasites [41] can negatively influence the body condition. Cautiously interpreted, our results may indicate there was appropriate food availability in the region and absence of overpopulation during the period of study.

Several species of ticks have been identified in MD and BBD, the most common being *Amblyomma* spp. and *Rhipicephalus microplus* [42–43]. Although not previously reported in MD and BBD, myiasis due to *Cephenemyia stimulator* and *Hypoderma diana* have been identified in other deer species [44]. Cat flea (*Ctenocephalides felis*) infestation in MD [45] and pediculosis in BBD [19] have also been reported. Ectoparasite characterization was not within the scope of this study; future studies may focus on characterizing ectoparasites in these species.

### Trauma in deer and anthropic origin

In wildlife, traumatic injuries may be induced by humans, *e*.*g*., poaching, fire arms, archery, vehicle, building-collision, barotrauma; or by animals [46–48]. Main causes of intraspecific trauma in deer involve interactions linked to a wide variety of factors (*e*.*g*., change of social status within the herd, overcrowding, fighting of bucks during the reproductive season, competition over limited feeder space), and vehicle collision and dog attack [49–51]. We observed a high occurrence of trauma, mainly associated with vehicle-collision and dog attack. Nevertheless, we believe trauma might have been underrepresented because these cases often are not subjected to postmortem investigations [52]. Vehicle-collision in BBD was mostly observed in adult animals with good body condition. Four of these animals had underlying disease, suggesting that preexisting pathologies may be a risk factor for vehicle-collision. Interestingly, some studies found no significant underlying disease in roadkill deer [53]. Postmortem examination of vehicle-collision cases may be of great value for health population monitoring [54–56]. In Brazil, it is estimated that around 15 animals die per second by vehicle-collision, accounting for more than 470 million animals annually [57]. These data are likely underestimated, since retrieval of corpses is likely incomplete and some traumatized animals may die off the road. Furthermore, dog attack was an important cause of death in BBD. Feral dog attack is considered one of the main causes of deer species admission to rescue and rehabilitation centers in Chile [58], and it is an important threat in the Chilean huemul (*Hippocamelus bisulcus*) [59] and Pudu (*Pudu* spp.) [60]. Although we did not find any association between trauma and age, some studies found fawns to be more predisposed [59, 61].

The multiple trauma-associated lesions observed in this study (contusions, extensive muscular and systemic hemorrhages including the spinal canal and CNS, abdominal puncture, and axial and appendicular fractures and luxations) are in agreement with previous studies [62–64]. Atlantoaxial luxation was observed in two MD with history of severe stress and self-induced trauma against a fence. Similar findings were described in deer due to fence collision [65].

### Alimentary tract, liver and pancreas

*Actinobacillus* sp. and *Mannheimia* sp. infections have been associated with purulent and necrotizing glossitis in deer [66–67]. Furthermore, necrotizing glossitis may be observed in BTV infection [68–69]. We observed some cases of necrotizing and hemorrhagic glossitis with concomitant multisystemic hemorrhage. Although not tested, orbiviral infection (EHDV, BTV) was considered a possible etiology.

In cattle, ruminal mycosis is often a consequence of acute disease, sudden increase in the ruminal load and lactic acidosis [70–71]. Ruminal candidiasis has been recorded in wildebeest [72], WTD [73] and American bison [74]. *Aureobasidium pullulans* is a black yeast-like species found in environments with fluctuating water activities [75]. Furthermore, *A*. *pullulans* has been associated with cutaneous and systemic infections in immunosuppressed humans [76–80] and occasionally ascribed to cutaneous infections in few animal species [81]. In the present case, the histomorphological features of the fungus and PCR analysis supported the diagnosis of ruminal mycosis by *A*. *pullulans*. A potential cause of immunosuppression or predisposing factor other than age was not readily evident in this case.

Portal hepatitis was the most common inflammatory pattern in the liver. Although an etiology was not readily evident, the main differential diagnoses would be unresolved chronic viral hepatitis and nonspecific reactive hepatitis. Among viral agents, one of particular interest for public health is hepatitis E virus, due to its zoonotic potential. This virus has been reported in free-living and captive deer species [82–85], and transmission through contaminated bushmeat has been confirmed [86–87]. Future studies should investigate the potential circulation of these agents in Neotropical deer. We observed pancreatic duct trematodiasis compatible with *Eurythrema* sp. in a BBD. This genus was previously described in Brazilian cattle [88–89] being *E*. *pancreaticum* the most frequently reported species [90–91]. One MD had esophageal nematodiasis compatible with *Gongylonema* sp. *Gongylonema* sp. have been widely described in white-tailed deer [92], but not in South American species. Furthermore, we observed diaphragm myositis with intralesional nematodes compatible with ascarids. Aberrant migration of nematode larvae may cause inflammation and myonecrosis, as observed in our case. The most important genuses are *Ancylostoma*, *Toxocara* and *Baylisascaris* [93], yet to be reported in deer. Further etiological identification in our case was not achieved. To the authors’ knowledge, no hepatic neuroendocrine neoplasm subtype has been reported in deer. This tumor subtype is relatively uncommon in domestic species, including dogs [94] and cats [95]. In our case, neoplastic cells failed to express chromogranin and synaptophysin. Prolonged tissue fixation in formalin in this case may have precluded IHC analysis. Tumor cells were also negative for AE1/AE3, CD3 and CD20. In this light, the cell origin could not be confirmed.

### Cardiovascular system

Fungal myocarditis is rare in cattle [96], and most reports involve zygomycetes and *Aspergillus* spp. [97]. In the present case, PCR analysis identified *Phaeosphaeria* sp. Although this genus comprises common phytopathogens such as *P*. *nodorum* [98], it has not been linked to disease in animals. Some plant fungi can cause infection in humans and animals [99–100]. *Phaeosphaeria* sp. is closely related to the animal pathogenic fungus *Alternaria* sp. [101], one of the agents responsible for phaeohyphomycosis [102]. In the present case, there are histomorphological and molecular evidences that suggest *Phaeosphaeria* sp. as a potential etiology of fungal myocarditis.

### Endocrine

In ruminants, suppurative adrenalitis is typically associated with bacterial septicemia, whereas toxoplasmosis and herpesviral lesions are more commonly necrotizing and lymphohistiocytic [103]. In most of these cases, an etiology was not readily apparent, except for one case where adrenalitis was associated with bacterial sepsis.

### Musculoskeletal system

Suppurative myositis is generally of bacterial origin, and may derive from hematogenous colonization or local inoculation by a perforating wound. In ruminants, the main bacteria involved are *T*. *pyogenes* and *Corynebacterium pseudotuberculosis* [104]. In the present study, BBD myositis cases often had history of feral dog attack, indicating direct inoculation by a perforating wound as the entry route. No bacterial cultures were attempted to identify the involved bacteria in our cases.

### Nervous

Intracranial abscesses are common and often fatal in North American deer. They are commonly associated with secondary bacterial infection after trauma or antler fracture, being more frequent in males. The main bacteria isolated from intracranial abscesses include *Trueperella pyogenes* and *Actinomyces pyogenes* [105–107]. Bacterial meningoencephalitis is also found in European deer with an apparent high incidence of *Listeria monocytogenes* [108]. In the present case, the etiology could not be determined.

### Respiratory system

Pneumonia is one of the most important ailments in cervid medicine and causes may include viruses, bacteria, fungi, metazoan parasites and physicochemical agents [109–112]. Pneumonia was the most frequent inflammatory process in both species. In deer, the most frequently isolated bacteria from pneumonic lung tissues are *Arcanobacterium pyogenes*, *Escherichia coli*, *Fusobacterium necrophorum*, *Klebsiella pneumoniae*, *Manheimia haemolytica*, *Mycobacterium* spp., and *Streptococcus gallolyticus* (syn. *S*. *bovis*) [109–112]. Bronchopneumonia was the most common pattern, commonly associated with cocci and coccobacillary bacterial aggregates. Although due to logistical limitations we were unable to perform bacterial culture, the morphological characteristics and gram staining properties of bacteria within inflammatory foci suggested that *Manheimia* spp., and *Streptococcus* spp., could possibly be involved in our cases.

Most common etiologies of pulmonary nematodiasis in deer include *Dictyocaulus* spp. and *Protostrogylus* spp. In these cases, proliferation of first stage larvae causes diffuse interstitial pneumonia, and together with multiple granulomas in the parenchyma are also common features of verminous pneumonia [113–115]. In this study, a considerable number of animals of both species had verminous pneumonia. The morphological features of adult nematodes and eggs were compatible with family Metastrongylidae, most likely *Dictyocaulus* spp. Pulmonary hydatid cist-like remnants suggestive of *Echinococcus* sp. were observed in one BBD. *Echinococcus granulosus*, a cestode with zoonotic potential, has been previously reported in deer [116]. Furthermore, *Taenia omissa* [117] has also been associated with lung cysts in deer. The etiology in this case could not be confirmed.

Lymphoplasmacytic perivasculitis may be observed in various infectious diseases of deer such as BVDV, *Mycoplasma bovis* [118], epizootic hemorrhagic disease [119] and malignant catarrhal fever [120–122]. BVDV is a well-known immunosuppressant and an important cause of bovine respiratory disease [123–126]. Diagnosis of BVDV has been attempted without success in South American deer species [18, 127]; nevertheless, seropositivity was detected in Colombian WTD [128]. In cattle, *M*. *bovis* infection may cause chronic bronchopneumonia with necrosis and bronchiectasis and occasionally fibrinous to fibrous pleuritis [129]. We investigated herpesvirus by PCR in a set of cases with lymphoplasmacytic perivasculitis; however, all tissue tested were negative. No other etiologies were investigated in these animals.

The etiology of pulmonary edema may be multifactorial, involving infectious (*e*.*g*., early stages of pneumonia, sepsis) and non-infectious causes (e.g., inhalation of smoke and fumes, anaphylactic reactions, plant or drug intoxication, trauma); however, it lacks specificity [130]. Pulmonary edema was the second most common finding in both deer species. Potential causes held responsible for pulmonary edema and moderate to marked lung congestion in this study were various and included severe tick infestation, severe starvation, trauma and pneumonia.

### Urogenital system

Focal lymphohistiocytic inflammation is common in kidneys of many animal species and causes are seldom known [131]. In ruminants, multifocal interstitial nephritis may occur in viral (*e*.*g*., MCF, lumpy skin disease, adenovirus), bacterial (e.g., *Escherichia coli*, *Salmonella* sp., *Brucella* sp.) and protozoal (e.g., *Theileria* sp.) infections [131]. In deer, interstitial nephritis has been linked to MCF [132–133], leptospirosis [134–135] and elaeophorosis [136]. Some animals with interstitial nephritis had additional lesions suggestive of MCF; however, molecular analysis was negative. No etiology was evident for the interstitial nephritis observed in these animals.

Dysgerminoma [137] has not been reported in deer, and is relatively uncommon in veterinary species, including cattle [138–139], equine [140–141], dog [142–143], and a snow leopard [144]. In dogs, neoplastic cells are mainly positive for vimentin and alkaline phosphatase, and negative for CD3, CD79α, cytokeratin, alpha-fetoprotein, inhibin-a and S-100 [145]. In the present case, the IHC panel was limited to vimentin and S-100. Although there are divergent results for this immunomarkers in this neoplasm subtype in different animal species and humans, we believe the anatomical location, cytological features and IHC findings support a diagnosis of dysgerminoma [146].

### Hemorrhagic disease

Systemic hemorrhage, predominately involving the lungs, kidney and liver, and less often the intestines, urinary bladder and gonads, was a relatively common finding in this study. For most of these cases, historical trauma would explain the hemorrhages. Hemorrhagic disease (caused by epizootic hemorrhagic disease virus) is a well-known infectious disease in deer, endemic in certain areas of Brazil [147–150]. The *Mazama* and *Blastocerus* genera are vulnerable [151]. After the bite of a contaminated insect, viral replication occurs in the regional lymph nodes and spleen. Viremia is then followed by widespread endothelial damage (microangiopathy), leading to hemodynamic disturbances such as endothelial swelling, thrombosis of small vessels, intravascular disseminated coagulation, edema and hemorrhage [67, 152, 153]. In the present cases, the clinical signs, gross, and microscopic findings were highly suggestive of epizootic hemorrhagic disease; however, molecular diagnosis could not be performed. Future studies are needed to characterize the geographic and host range of orbiviral diseases in Brazil along with comparative pathological studies to assess potential species susceptibilities to orbivirus infection.

### Causes of death

Trauma is commonly reported at varying rates (19% to 52%) in both free-ranging and captive deer [154–155], yet they are less common in captive conditions [63, 155]. Death by trauma in these species may result from one or more of the following events: 1) cranioencephalic or spinal cord trauma leading to severe CNS damage and subsequent cardiorespiratory collapse; b) hemorrhagic shock caused by external or internal bleeding; and c) transdiaphragmatic herniation of the liver and the polygastric complex with subsequent pulmonary compression and asphyxia [64]. In this study, trauma was the leading cause of death in BBD and the second in MD. Cranioencephalic and/or spinal cord trauma and hemorrhagic shock prevailed in the present study. Noteworthy, the main causes of trauma were anthropogenic such as vehicle-collision and feral dog attack.

Comprehensive studies on pathological findings, morbidities and mortality in North American and European deer are currently available in the literature. By contrast, analogous large-scale and long-term studies on South American deer are very limited [155]. Cause of death (CD) in Argentinean MD included: high parasite burden, unusual adverse climatic conditions and apparent reduction in the availability of pastures [156]. In this study, the most important CD in MD and BBD involved respiratory disturbances, as observed in free-ranging and captive North American deer populations [62–63]. Starvation has been indicated as a predisposing death factor in wild European and North American deer populations [157–160]. However, our results failed to support starvation as a significant morbidity and mortality factor in MD and BBD. Other identified CD included: enterocolitis, gastrointestinal parasitism, myopathy and nephritis [62, 63, 154, 161].

## Supporting information

S1 Table Tissues evaluated by species. (XLSX)

S2 Table Epidemiological, biological data, gross and microscopic findings, and most probable causes of death. NR = No reported.
(XLSX)
